# Formation Mechanism of Dilute Region and Microstructure Evolution in Laser Solid Forming TA15/Ti_2_AlNb Dual Alloy

**DOI:** 10.3390/ma13030552

**Published:** 2020-01-23

**Authors:** Hua Tan, Zesen Mi, Yongshuai Zhu, Zhenyu Yan, Xin Hou, Jing Chen

**Affiliations:** 1 State Key Laboratory of Solidification Processing, Northwestern Polytechnical University, Xi’an 710072, China; mizesen@mail.nwpu.edu.cn (Z.M.); zhuys@mail.nwpu.edu.cn (Y.Z.); houxinn1@mail.nwpu.edu.cn (X.H.); 2 Capital Aerospace Machinery Co., Ltd., Beijing 100076, China

**Keywords:** laser solid forming, TA15/Ti_2_AlNb dual alloy, dilute region, microstructure evolution

## Abstract

TA15/Ti_2_AlNb multiple-layer samples and a dual-alloy sample were fabricated by laser solid forming (LSF) in this study. The formation mechanism of the dilute region and microstructure evolution of the dual alloy were analyzed. The results confirmed a “step” distribution of the composition among several initial layers in the multiple-layer samples, which can be explained by calculating the ratio of the remelted zone to the deposited Ti_2_AlNb zone in each deposited layer. However, the “step” compositional distribution disappears, and the compositional variation tends to be more continuous and smooth in the TA15/Ti_2_AlNb dual-alloy sample, which is attributed to alloy elements’ diffusion at the subsequent multiple re-melting and the longer thermal cycle. The macrostructure of the TA15/Ti_2_AlNb dual-alloy sample consists of epitaxially grown columnar prior β grains at the TA15 side and equiaxed grains at the Ti_2_AlNb side, while the microstructure shows a transition of α+β→α+α_2_+β/B2→α_2_+β/B2→α_2_+B2+O with increasing amounts of Ti_2_AlNb, leading to the microhardness also changing significantly.

## 1. Introduction

Laser solid forming (LSF) is an advanced material-forming technology that can be used to fabricate high-performance 3D near-net shape metal components without the need for tooling [1,2]. Samples were deposited point-by-point, line-by-line, and layer-by-layer via the melting/solidification of metal powders. This principle of metal forming offers distinct advantages over conventional manufacturing methods for the manufacture of graded-gradient materials or dual alloys with spatial variance in the context of chemistry and microstructure [3]. As a branch of functional-graded materials, dual alloys are primarily used as insulation structural materials in the aerospace industry. As a widely used titanium alloy in the aerospace industry, TA15 (Ti-6.5Al-2Zr-1Mo-1V wt %) is reported to have mid-level strength at both extreme and room temperatures, excellent weldability and heat endurance, as well as the ability to withstand extended periods of usage at 500 °C [4]. The Ti_2_AlNb (Ti22Al25Nb at %) intermetallic alloy is utilized at 650–850 °C due to its outstanding specific strength, prominent high-temperature performance, and its low coefficient of thermal expansion [5,6,7]. Recent research focused on Ti_2_AlNb in the context of selecting suitable alloys and integral formation that satisfy requirements for the low weight and working temperature of structural materials in the aerospace industry. The fabrication of a TA15/Ti_2_AlNb dual alloy is feasible [8,9], since Ti_2_AlNb intermetallic alloys can be used at the windward side of the rudder wing on an aerospace craft, where it is exposed to high working temperatures, while TA15 can be utilized at the positions where the working temperature is lower to decrease the total weight of the component.

Since the advent of LSF for manufacturing metallic components, many research studies have focused on the manufacture of functionally graded materials (FGM) or dual alloy. Liang et al. [10] fabricated Ti/Ti-6Al-2Zr-1Mo-1V graded material and systematically elucidated its compositional variation, microstructural evolution, and the mechanical behavior of the dual alloy. The results showed a gradual change of alloying elements at the interface due to the diffusion from the heat effect of the subsequent deposition process. Savitha et al. [11] compared the composition variation and mechanical properties of laser-engineered net shaping (LENS) SS316-Inconel 625 dual alloy and graded material, and they pointed out that the elemental composition exhibited gradual changes near the transition region of the graded materials. In addition, a discrete change near the interface of the dual alloy was also evident, which contradicted Liang’s result. Unocic et al. [12] analyzed the effect of the LENS process parameters on the dilution and compositional variation in FGMs, and reported that the dilution due to substrate re-melting is directly proportional to both laser power and scanning speed, while being inversely proportional to the powder feed rate. Liu et al. [13] studied the dilution of Al and V in the Ti/Ti6Al4V graded material during the laser powder deposition (LPD) process. Convection and stirring in the molten pool and the subsequent thermal diffusion resulted in gradual elemental content changes. Bobbio et al. [14] used the directed energy deposition (DED) to form linearly graded Ti-6Al-4V/Invar 36 FGM; however, the samples prepared in his work had macroscopic cracks, which were assumed to be caused by the mismatches of the secondary phases in thermal expansion and elastic modulus. Shang et al. [15] fabricated a TA15-Inconel 718 bimetallic structure via Nb/Cu multi-interlayers via laser additive manufacturing, and reported that Ti, Nb, Cu, and Ni elements diffused due to the heat effect of the laser and high-temperature gradient during the deposition process, which effectively prevented the formation of brittle phases while also strengthening the metallurgical bonds between both alloys.

Despite the plethora of research on the composition variation and formation mechanism of the dilute region (or transition zone), a consensus on the subject of elemental variation within the dilute region is noticeably absent, which makes further research into this matter prudent. Microstructural evolution is strongly reliant on elemental distribution and directly influences the resulting mechanical properties of the formed alloys. In this study, TA15 and Ti_2_AlNb alloy powders were used to fabricate LSF dual-alloy samples, and two experiments were conducted to elucidate the dilute region-forming mechanism and microstructural evolution of the LSF dual alloy.

## 2. Experimental Procedures

**Figure 1:**
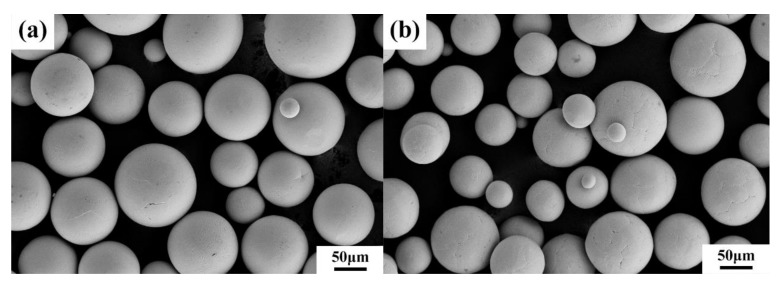
The SEM pictures of powder particles of TA15 and Ti_2_AlNb: (**a**) TA15; (**b**) Ti_2_AlNb.

Pre-alloyed TA15 and Ti_2_AlNb spherical powders produced by the plasma rotating electrode process (PREP) were used as the deposited material. The spherical shapes of particles of TA15 and Ti_2_AlNb powders are presented in Figure 1. The powder sizes were from 45 to 180 μm, and the compositions determined by inductive coupled plasma emission spectrometer (ICP) are listed in Table 1. Before fabrication, the powders were dried in a vacuum oven for 2 h at 400 K to eliminate moisture absorption and ensure the liquidity of the powders. The forged TA15 plates, with dimensions of 140 mm × 50 mm × 25 mm, were used as substrates, and the depositing surfaces were grounded with SiC paper and then degreased with acetone and ethanol before being used. The samples used in this study were formed using the LSF-S6000A equipment, which consists of a 6 kW continuous semiconductor laser unit, a five-axis numerical control working table, and a coaxial powder feeder with four nozzle tips. The experiments were carried out in an inert gas chamber filled with pure argon (Ar), with oxygen (O_2_) content below 50 ppm to prevent the occurrence of oxidation.

**Figure 2:**
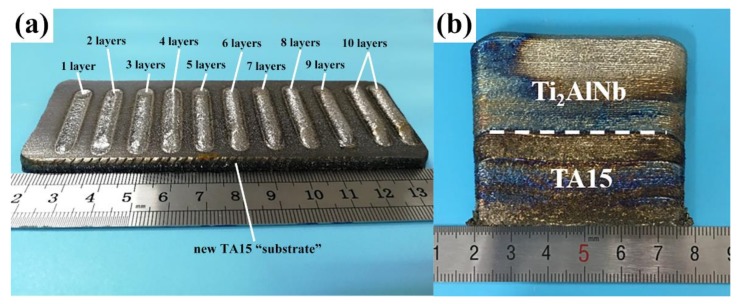
Samples fabricated by laser solid forming (LSF): (**a**) multiple-layer samples fabricated in Experiment 1 (E1); (**b**) dual-alloy sample fabricated in Experiment 2 (E2).

Two sets of LSF experiments were designed in this study. Experiment 1 (E1) is the deposition of the multiple-layer samples, which was designed to exploit the formation mechanism of the dilute region in the TA15/Ti_2_AlNb dual alloy and determine the composition formation regularity of each of the newly deposited layers. A TA15 bulk, with dimensions of 140 mm × 50 mm × 10 mm, were first fabricated on the forging TA15 substrate; then, the deposited bulk was flatten by a wire-cut electrical discharge machine, polished with abrasive paper, and acted as a new “substrate”. Then, Ti_2_AlNb deposition was conducted by 1 layer, 2 layers, …, 10 layers respectively on the new TA15 “substrate”. Finally, 1–10 layers of Ti_2_AlNb single-wall samples are completely shown in Figure 2a.

Experiment 2 (E2) is the deposition of TA15/Ti_2_AlNb dual alloy, which was done to study the microstructure evolution and composition transition at the dilute region of the TA15/Ti_2_AlNb dual alloy. Single-wall TA15 samples with heights of ~30 mm was deposited and used as the “substrate”; then, the same height (~30 mm) of Ti_2_AlNb was deposited onto LSF TA15. A dual-alloy sample with dimensions of 60 mm × 5 mm × 60 mm was fabricated. The processing parameters of both experiments were chosen as a laser power of 2 kW, a spot diameter of 5 mm, a scanning speed of 10 mm s^−1^, a powder feed rate of 11 g min^−1^, a *Z* increment ∆*Z* of 0.3 mm, and an overlapping ratio of 40% (just for E2). The deposited sample is shown in Figure 2b. In order to eliminate the thermal stress generated during the LSF processing, 550 °C/2 h annealing heat treatment was conducted using the SX-4-10 box-type electrical resistance furnace. Olympus-PMG3 and Keyence VH-Z50L light microscopy (LM) were used to image the macrostructures of the samples. The TESCAN VEGALMH Scanning Electron Microscope (SEM), equipped with an energy-dispersive spectroscopy (EDS), was used to determine the microstructure in the prior β grains and the elemental distribution in the deposited samples. The metallographic samples were cut from the deposited samples and etched using the Kroll etchant (H_2_O:HF:H_2_O_2_:HNO_3_ = 20:2:3:7).

## 3. Results and Discussion

### 3.1. Microstructure and Compositions of Multiple-Layer Samples

**Figure 3:**
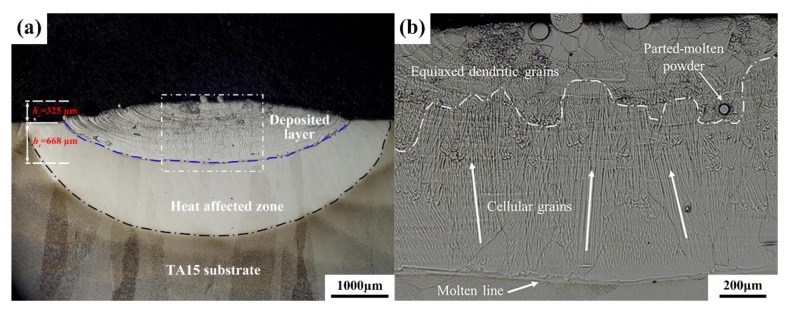
The light microscopy pictures of the cross-section of the first deposited layer in E1: (**a**) macromorphology; (**b**) magnified image of the microstructure in the deposited layer.

Figure 3 shows the macromorphology of the cross-section of the laser-deposited single layer from the E1 experiment. It can be seen that the metallographic sample is made up of three regions: the deposited layer, the heat-affected zone, and the substrate zone. It can also be seen from the magnified image of the microstructure at the mid-deposited layer that the primary solidification microstructure is duly retained. The entire deposited layer can be divided into two parts based on its respective grain morphologies: the equiaxed grains region at the top of the deposited layer and the columnar grains region with the cellular substructures at the middle and bottom of the layer.

**Figure 4:**
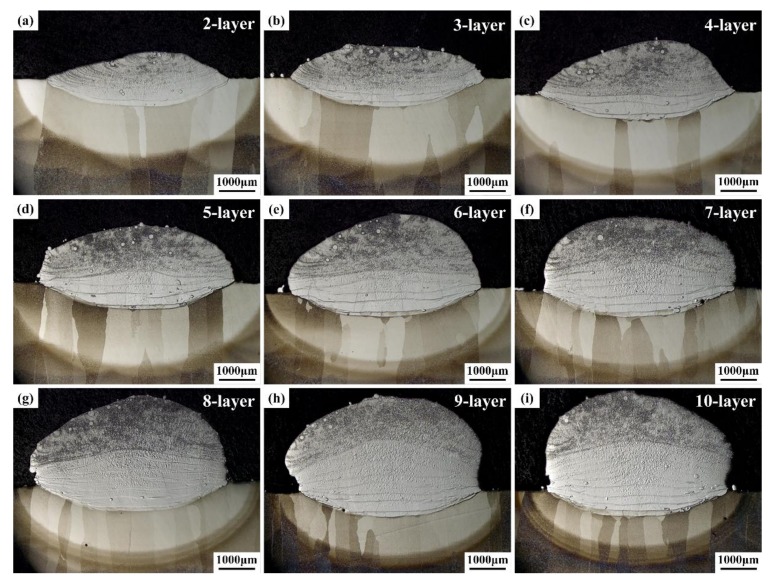
Cross-sections of multiple-layer samples on TA15 substrate: (**a**) the two deposited layers; (**b**) the three deposited layers; (**c**) the four deposited layers; (**d**) the five deposited layers; (**e**) the six deposited layers; (**f**) the seven deposited layers; (**g**) the eight deposited layers; (**h**) the nine deposited layers; (**i**) the ten deposited layers.

Figure 4 shows the macrostructure of the other single-wall samples in E1. As per Figure 4, the melting lines are evident at the bottom of each sample, with each consisting of the heat-affected and dilute regions in the middle and the deposited layer region at the top. It can also be seen that the original dendritic structure was retained.

**Figure 5:**
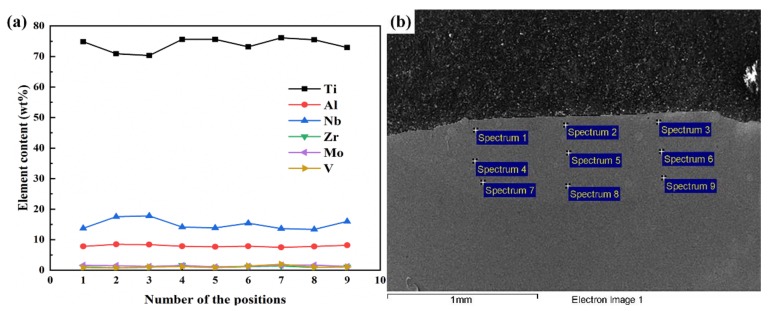
Chemical compositions of the single-layer sample in E1: (**a**) element content of each position in the deposited layer; (**b**) energy-dispersive spectroscopy (EDS) testing positions in single layer sample.

Figure 5a presents the EDS results of nine points at multiple regions of the first laser-deposited Ti_2_AlNb layer on the LSF TA15 substrate, and the tested positions are shown in Figure 5b. As per Figure 5, the elements are basically homogeneously distributed within the first deposited layer, as the difference of the main element Ti content between the top and bottom regions is <5.3 wt %.

**Figure 6:**
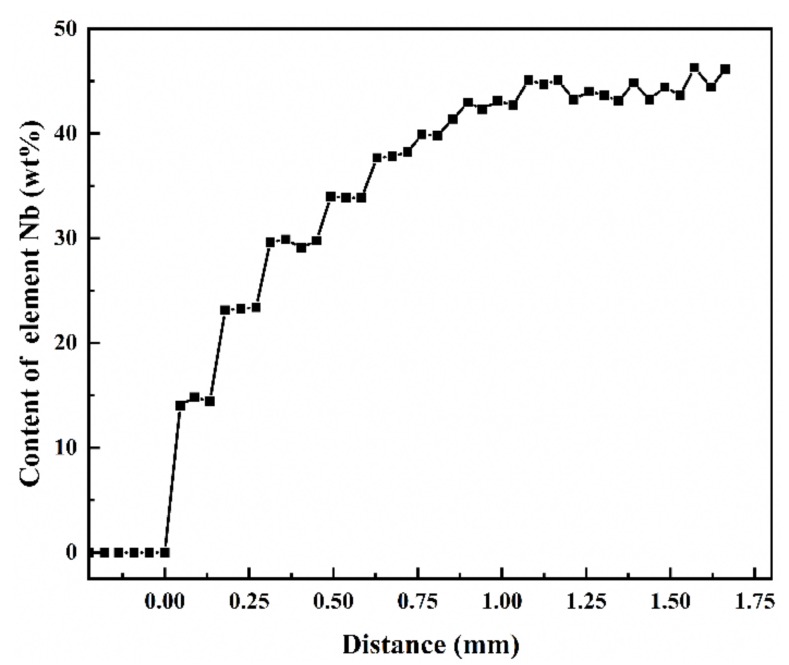
The Nb content vs. the distance from the molten line of the 10-layer sample.

Table 2 shows the main elemental (Ti, Al, Nb) compositions within the last deposited layer of each sample in E1. As per Table 2, from the compositions on top of the single-layer sample to the 10-layer sample, it was found that the elemental compositions transited to Ti_2_AlNb until the ninth layer, while the elemental compositions of the first to eighth layers were in the range of TA15 to Ti_2_AlNb. In order to determine the regular elemental distribution, the distribution of Nb within the 10-layer sample is shown in Figure 6, as it was measured along the central line of the cross-section shown in Figure 4i, and the intersection of the center line and molten line was set as “0”. As per the plot, an obvious composition “step” is evident among the first few layers. As the plot curve becomes continuous, the composition “step” disappears after several layers.

**Figure 7:**
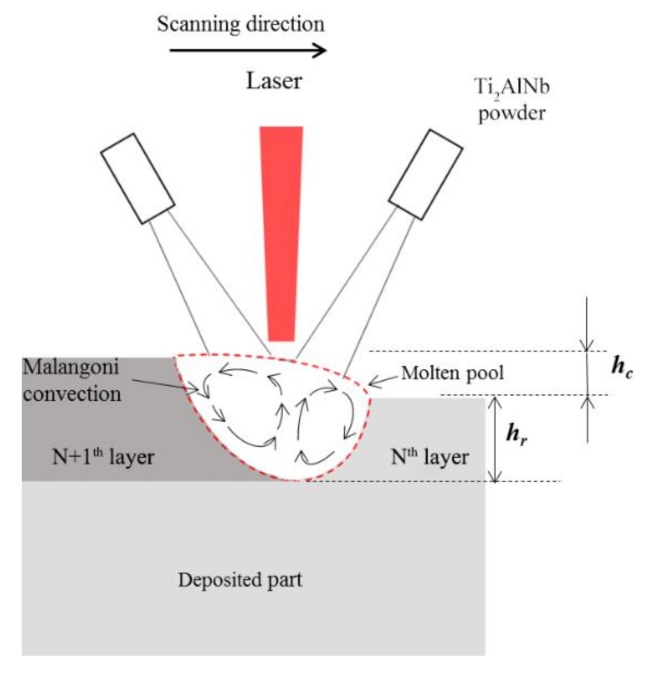
The schematic of the dilute effect in the molten pool.

During LSF, the alloy powders were melted on the surface of the deposited layers using a high-power laser beam, forming a metal pool. According to the Marangoni effect by Anthony and Cline [16], surface tension and buoyancy are the two main forces present within the molten pool, and the forced convection of metal liquid flow is caused by the surface tension gradient. It is also known that the significant surface tension gradient that originates from the center to the margin of the pool is present. The temperature of the metal flow right under the laser beam is high, while its surface tension is low. Moving further from the center of the laser beam, the temperature of the metal flow decreased, which formed a tension gradient. Due to the presence of this gradient, the metal liquid on the surface of the molten pool flow from the center to the margin, forming an altitude intercept at the liquid metal. The gravity gradient caused by this altitude intercept resulted in a back flow of liquid metal, forming two symmetrical circulation loops of liquid metal flow (Figure 7).

In E1, when depositing the N+1th layer, the upper part of the solidified Nth layer was re-melted, and the Ti_2_AlNb powder particles fed to the molten pool were melted by a high-power laser beam, which initiated the Marangoni convection, due to the circulation convection of the element homogeneously mixed within the molten pool. Figure 7 shows that the elemental composition of the N+1th layer is intermediate between the Nth layer and Ti_2_AlNb powder due to the mixing elements in the molten pool.

**Figure 8:**
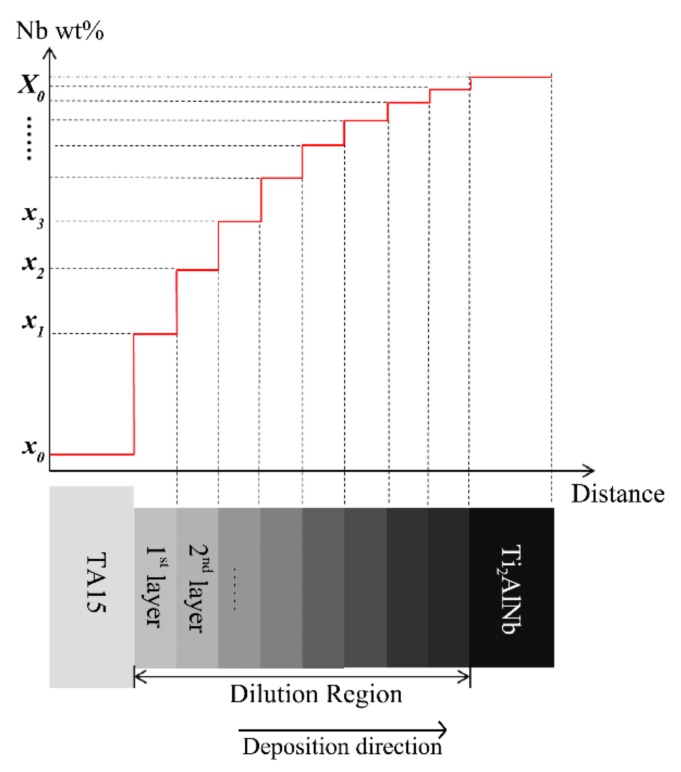
The schematic of the forming mechanism of the dilute region in the LSF TA15/Ti_2_AlNb 10-layer deposited sample.

Figure 8 displays the dilute region-forming mechanism of the LSF dual alloy or graded materials. During the process of depositing the first layer, deposited TA15 (elemental composition, *x*_0_) was re-melted, Ti_2_AlNb powder particles (elemental composition, *X*_0_) were fed to the molten pool, and the two powders were sufficiently mixed. As shown in Figure 7, *h_c_* and *h_r_* represent the deposited and re-melted heights, respectively; therefore, the practical composition of the molten pool can be decided by *h_c_* and *h_r_.* For example, the composition of the first layer *x*_1_ should be:

(1) $$x_{1}=\frac{\left(h_{r}\times x_{0}+h_{c}\times X_{0}\right)}{h_{r}+h_{c}}.$$

According to the cross-section of the first deposited layer (Figure 3a), *h_c_* ≈ 325 μm, *h_r_* ≈ 668 μm; so, the composition of the first deposited layer can be obtained by

(2) $$\frac{h_{r}}{h_{c}}\approx 2$$

(3) $$x_{1}\approx \frac{\left(2x_{0}+X_{0}\right)}{3}.$$

Since the mass fraction of Nb was 44.12 wt % in the Ti_2_AlNb powder (Table 1), the content of Nb in the first deposited layer was calculated to be 14.71 wt % by using Formulas (1)–(3), which is very close to the test results in single deposited layer shown in Table 2 (13.97 wt %). In addition, the theoretical value of the elemental fraction in each layer can be calculated by

(4) $$\begin{array}{c} x_{2}=\frac{\left(2x_{1}+X_{0}\right)}{3}\\ x_{3}=\frac{\left(2x_{2}+X_{0}\right)}{3}\\ \vdots \\ x_{n+1}=\frac{\left(2x_{n}+X_{0}\right)}{3} \end{array}.$$

The theoretical distribution of Nb, whose change was the most significant at the dilution region, is depicted in Figure 8. It is evident that the theoretical distribution of Nb in each deposited layer is consistent with the measurement results in the 10-layer deposited sample (Figure 6).

### 3.2. Microstructure Evolution in the Dilute Region of LSF TA15/Ti_2_AlNb Dual Alloy

**Figure 9:**
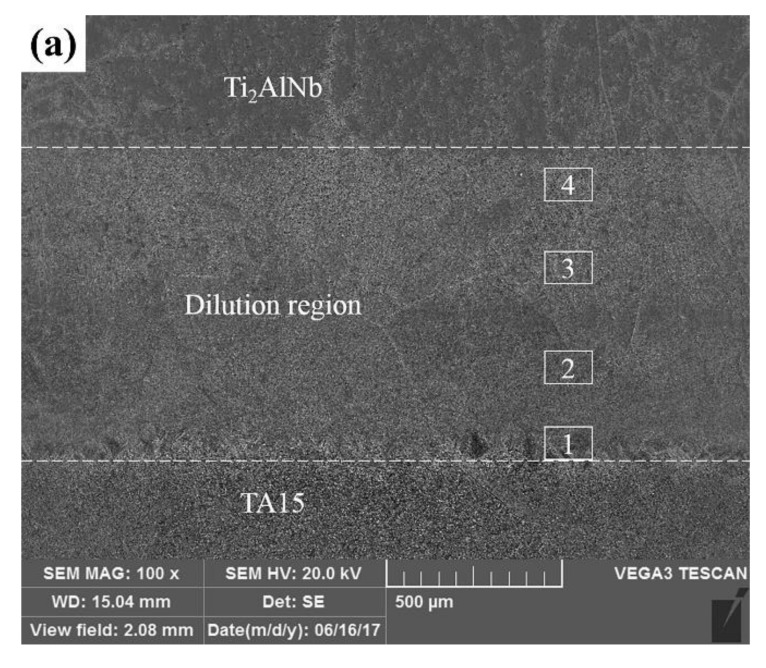

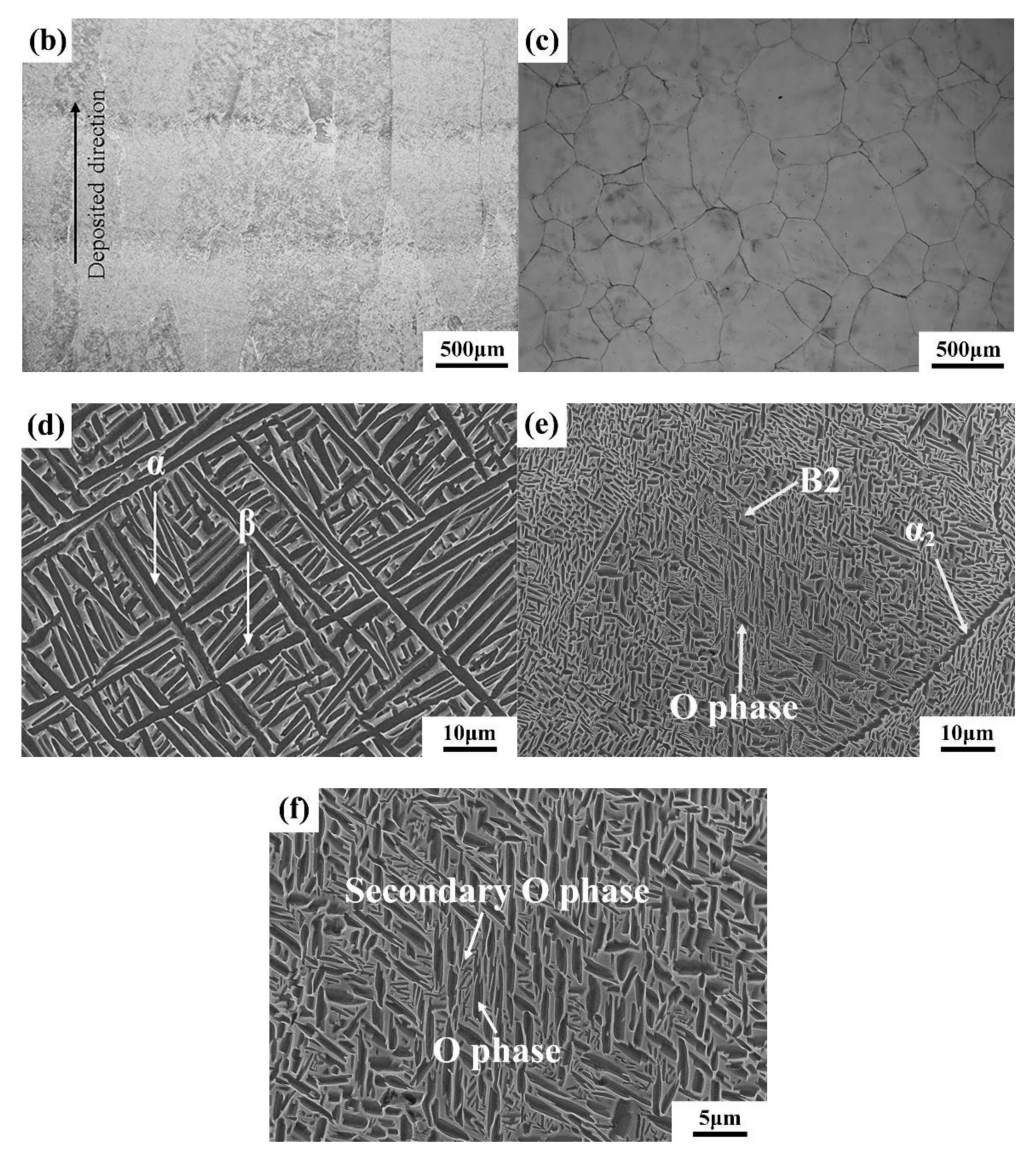
Microstructure of TA15/Ti_2_AlNb dual alloy: (**a**) the macromorphology of the microstructural distribution in the TA15/Ti_2_AlNb dual alloy; (**b**) the morphology of the prior β grains in the TA15 side; (**c**) the morphology of prior β grains in the Ti_2_AlNb side; (**d**) the microstructure in the β grains of TA15; (**e**) the microstructure in the β grains of Ti_2_AlNb; (**f**) highly magnified microstructure in the β grains of Ti_2_AlNb.

Figure 9a presents the macromorphology of the LSF TA15/Ti_2_AlNb dual alloy fabricated in E2. The macrostructure and microstructure of both sides of the TA15/Ti_2_AlNb dual alloy are shown in Figure 9b–e. The macrostructure at the TA15 side mainly contains coarsened columnar prior β grains that grow epitaxially via multiple deposited layers from the substrate. The length of the β grains is within 500–2000 μm, averaging to approximately 300 μm width (Figure 9b). The macrostructure at the Ti_2_AlNb side comprised equiaxed grains that had an average diameter of approximately 300 μm, as shown in Figure 9c. The microstructure in the prior β grains of TA15 consists of basket-weave α laths with an average width of approximately 1 μm and retained β distributed between α laths, as shown in the SEM images of Figure 9d. The microstructure in the prior β grains of the Ti_2_AlNb side is shown in Figure 9e. Relative to the TA15 titanium alloy, the microstructure of Ti_2_AlNb was much finer. It is evident that the black α_2_ phase precipitated along the grain boundary, and the rod-like O phase precipitated at the matrix of B2 phase. The length of the O phases is about 1–6 μm, approximately averaging to 0.7 μm width. It should also be pointed out that finer acicular secondary O phases are evident in the highly magnified SEM image, as shown in Figure 9f.

**Figure 10:**
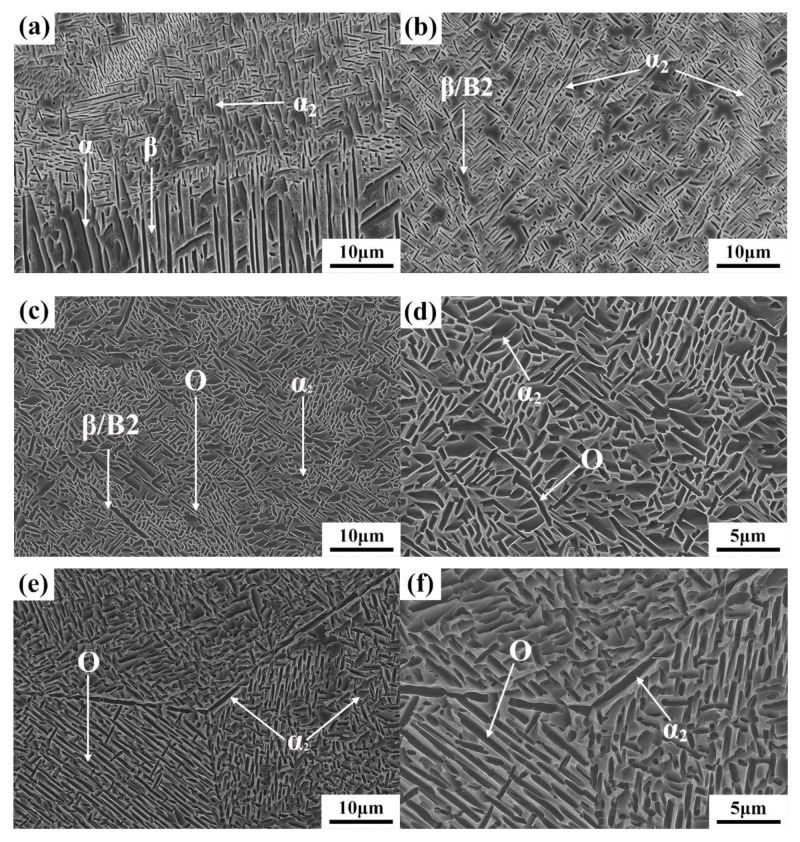
The microstructural evolution of the dilution region of TA15/Ti_2_AlNb dual alloy: (**a**) microstructure evolution of Region 1; (**b**) microstructure evolution of Region 2; (**c**) microstructure evolution of Region 3; (**d**) highly magnified microstructure evolution of Region 3; (**e**) microstructure evolution of Region 4; and (**f**) highly magnified microstructure evolution of Region 4.

**Figure 11:**
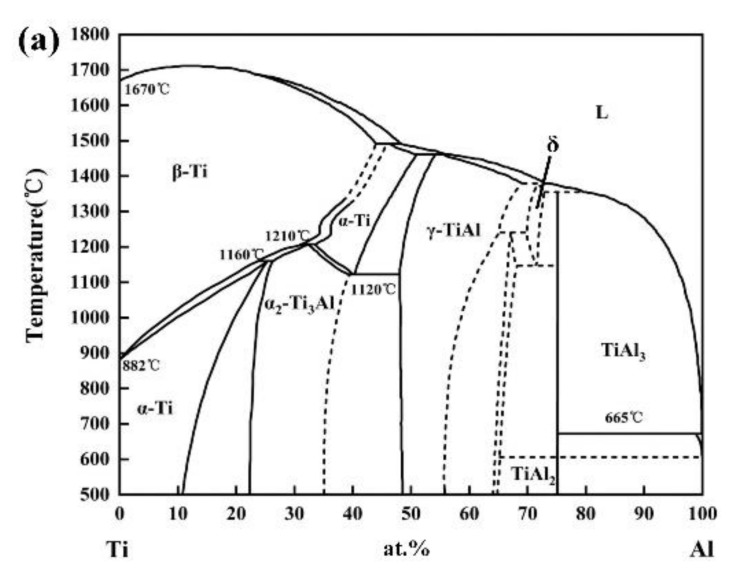

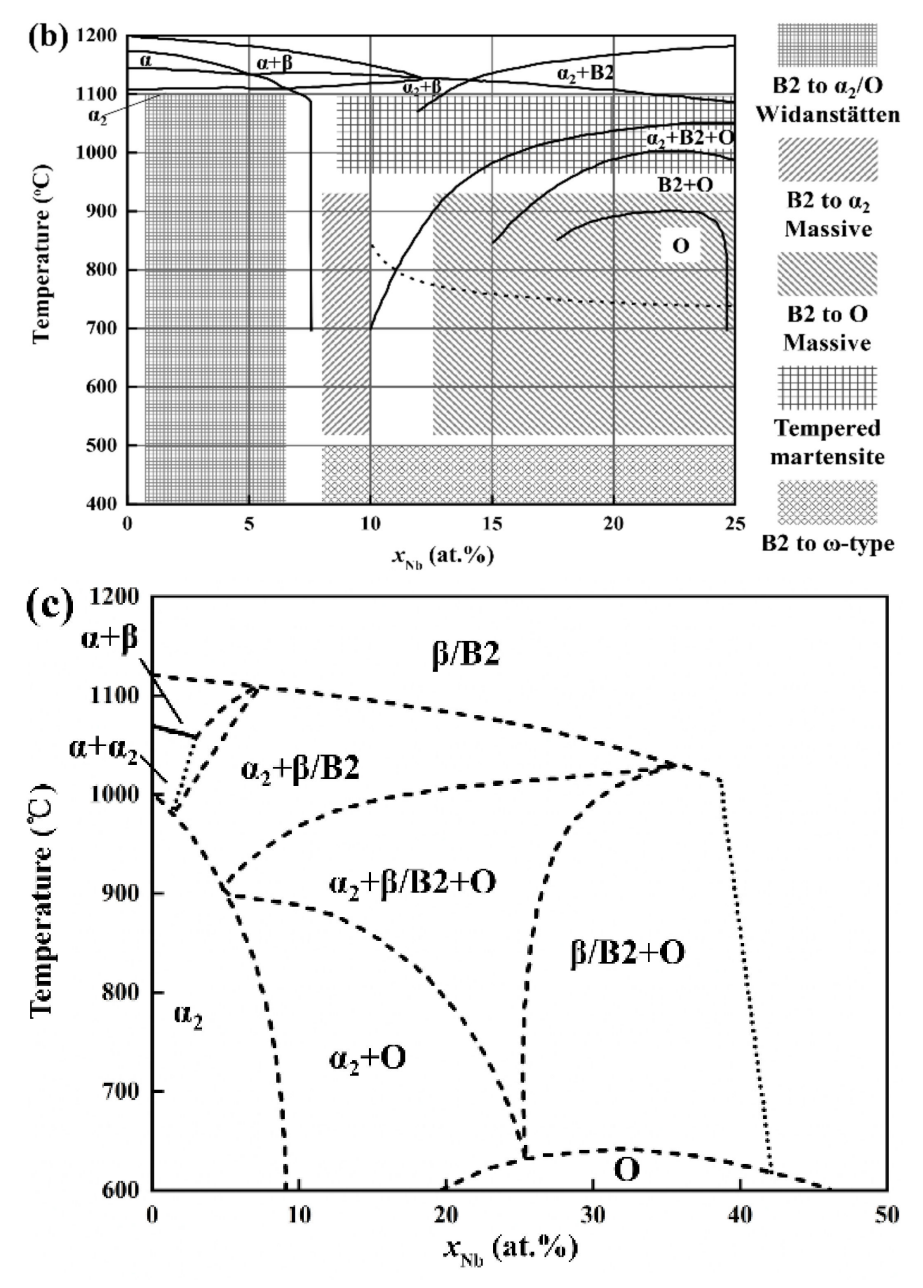
Phase diagrams used in this study: (**a**) Ti-Al binary phase diagram [17]; (**b**) Ti_3_Al-*x*Nb pseudo-binary diagram [18]; and (**c**) Ti-22Al-*x*Nb pseudo-binary diagram [19].

Figure 10 shows the microstructure of the dilute region of the LSF TA15/Ti_2_AlNb dual alloy. The dilute region can be divided into four parts based on its microstructures, as per the white square lines seen in Figure 9a. The microstructure at “Region 1”, which is near TA15, is shown in Figure 10a. Significant differences between the microstructure at Region 1 and TA15 are evident: the α laths at the bottom of “Region 1” transformed into a short rod-like α phase, while its width and fraction decreased. It should be pointed out that the α phase at this region tends to be parallel to the direction of the deposition, which is mainly due to the increasing Nb content (β stabilized element) preventing the precipitation of the α phase. The finer precipitated phase can be seen at the top of “Region 1”. According to the Ti–Al binary phase diagram [17] (Figure 11a), as the Al content in titanium alloy exceeds 12 at %, the α_2_ phase (Ti_3_Al) would precipitate. The Al equivalent of the TA15 titanium alloy is high, and the accretion of the Ti_2_AlNb alloy enhanced the Al equivalent in the dilute region. The EDS results also shows that the Al content reached 13.99 wt %. Although the EDS method is a qualitative analysis, it can lead us to assume that the precipitated phase at the top of “Region 1” is the α_2_ phase. Figure 10b shows the microstructure of “Region 2”. The Ti content is 65.82 wt %, while the Al and Nb contents are 16.38 wt % and 14.06 wt %, respectively, as per the EDS. Increasing the content of Al and Nb resulted in the precipitation of the α_2_ phase, while the matrix begins the transformation from the disordered β phase to the ordered B2 phase (the ordered phase of the β phase). Two morphologies of the α_2_ phase are evident: the strip α_2_ phase measuring 5–10 μm and a rod-like α_2_ phase that preferentially precipitates at the hypo-grain boundaries. According to the theory of the α_2_ phase precipitation, as the content of Al increases, the precipitation temperature of the α_2_ phase increases, and the precipitation resistance increases. Therefore, some α_2_ phases tend to precipitate preferentially along the sub-grain boundaries. Moreover, according to the Ti_3_Al–Nb pseudo-binary diagram [18] shown in Figure 11b, when the Nb content in the alloy surpasses 12 wt %, the matrix begins transforming from the β phase to the B2 phase. The EDS results of this region show that the Ti:Al ratio approaches 3:1, and the content of Nb is 14 wt %; therefore, the matrix may be the B2 phase in this region. Figure 10c shows the microstructure at “Region 3”. The EDS results show that elemental contents are 58.21 wt % Ti, 20.64 wt % Al, and 21.08 wt % Nb, which are similar to that of the Ti_2_AlNb intermetallic alloy. Combining the Ti-22Al-*x*Nb pseudo-binary diagram [19] (Figure 11c) led us to conclude that the microstructure at “Region 3” consists of α_2_+O+β/B2. Relative to “Region 2”, the higher Al content resulted in increased resistance for the α_2_ precipitation at “Region 3”, which decreases the volume of the α_2_ phase. It should be noted that the dispersive O phase (Ti_2_AlNb) precipitated in the matrix. As shown in Figure 10d, the fine rod-like O phase dispersed and distributed in the β/B2 matrix. Figure 10e shows the microstructure at “Region 4” being similar to a typical Ti_2_AlNb microstructure, which is always made up of the α_2_+O+β/B2 phase. Differing from “Region 3”, α_2_ phase tends to precipitate on the grain boundaries in the form of a mass of acicular, while rod-like O phase precipitated within the grains, which is mainly due to the increasing Al and Nb contents increasing the width of the α_2_+O+β/B2 three-phase region (Figure 11c), implying that more O phase precipitated during solidification, as shown in Figure 11f. The results of microstructure evolution are similar to Chen’s research [9].

### 3.3. Composition Distribution at the Dilution Region of LSF TA15/Ti_2_AlNb Dual Alloy

**Figure 12:**
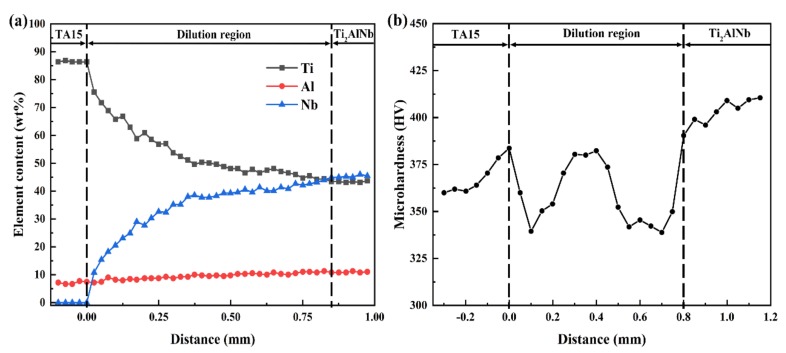
Composition and microhardness distribution at the dilute region of LSF TA15/Ti_2_AlNb dual alloy: (**a**) composition; (**b**) microhardness.

The main alloy elements (Ti, Al, Nb) within the dilute region of the TA15/Ti_2_AlNb dual-alloy sample deposited in E2 were also analyzed by using EDS, and the results are shown in Figure 12a. A dilute element with a width of approximately 800 μm can be seen in the image. The content of Nb increased while that of Ti decreased, and the content of Al increased slightly within the dilute region. It should be pointed out that the contents of Ti and Nb change exponentially and continuously, and the “step” characteristic was absent. One of the most significant characteristics of the LSF is that the deposited layers would undergo cyclic heat treatment [20]. Both finite element modeling and the temperature field obtained via direct observation of the LSF demonstrated that the temperature of each point experienced a process of quick fluctuation when exposed to the laser beam reciprocate [21,22]. When fabricating the Nth layer, the enormous amount of heat from the molten pool would transmit to the previously deposited layers, and as per the temperature field and element of diffusion laws, the diffusion ability and velocity of the alloy element is higher when its temperature is high. Compared with multiple layers, the dilute region of the dual alloy undergoes increased cycle heat treatment of the latter depositing layers; therefore, the alloying element, such as Ti, Al, and Nb could be distributed more homogenously. The diffusion of the alloying element results in a homogeneous and continuous distribution of the element, and the composition “step” disappears in the dilute region of the dual-alloy sample.

The change in the microhardness of the LSF TA15/Ti_2_AlNb dual alloy is shown in Figure 12b. In general, the change of hardness is complicated. There exists a secondary peak and two valleys at the dilution region. The microhardness values near the dilution region of TA15 were higher than those of the TA15 matrix, and gradually increased to 384HV along the deposition direction, mainly because the martensite α′ phase precipitated from the heat-affected zone changed to the fine basket-weave structure consisting of the α phase and β phase. The closer to the dilution region, the finer the phases. After entering the dilution region, the microhardness gradually decreased to 338HV. With the increasing of the content of the β stabilized element Nb, the size and quantity of the α phase decreased, resulting in the decreasing of microhardness. After that, the microhardness gradually increased and reached a maximum value of 385HV at the dilution region. The increasing of microhardness in this region was mainly because of the increasing of the content of Al and Nb, which enhanced the effect of solid solution strengthening. In particular, when Al content is higher than 12 at %, the precipitation of a hard brittle α_2_ phase leads to a significant increase of microhardness. Subsequently, because of the rapid increasing of the Nb element content, the stabilizing effect of Al on the α phase was greatly weakened, causing that only a small amount of the α_2_ phase precipitated along the grain boundary. The soft B2 phase was the main phase in the microstructure, so the hardness continues to drop to 339HV, even if a few of O phase precipitated. Later, the microhardness increased gradually mainly because the components gradually transferred to Ti_2_AlNb. Due to the precipitation of the O phase and α_2_ phase, the microhardness increased and reached 412HV at the Ti_2_AlNb region.

## 4. Conclusions

Perfect and dense TA15/Ti_2_AlNb multiple-layer and dual-alloy samples were fabricated using LSF. The forming mechanism and microstructural evolution of the dual-alloy dilute region were elucidated using the LM, SEM, and EDS techniques. The results led to the following conclusions:

(1) Elemental variation along the deposited direction of both the multiple-layer and the dual-alloy samples are characteristically exponential in its distribution; however, the composition “step” is evident in the multiple-layer samples. Compositional variation tends to be more continuous and smooth in the dual-alloy sample.
(2) The forced cycle convection of liquid metal caused by the Marangoni effect resulted in a homogenous mix of elements in the molten pool. The composition of each deposited layer is intermediate between the previous layer and the fed powders.
(3) The macrostructure of the samples consists of epitaxially grown columnar prior β grains at the TA15 side and equiaxed grains at the Ti_2_AlNb side. The microstructure evolution of the LSF TA15/Ti_2_AlNb dual-alloy dilute region exhibited a transition of α+β→α+α_2_+β/B2→α_2_+β/B2→α_2_+β/B2+O→α_2_+B2+O along the deposited direction. The microhardness also changed significantly with the transition of the microstructure.

## Figures and Tables

**Table 1:** Chemical compositions of TA15 and Ti_2_AlNb powders.

Material	Chemical Compositions, wt %								
	Al	Zr	Mo	V	Nb	N	H	O	Ti
TA15	6.65	2.12	1.79	2.23	--	0.008	0.024	0.13	Bal.
Ti_2_AlNb	11.10	--	--	--	44.12	0.005	0.002	0.08	Bal.

**Table 2:** Chemical compositions of the last deposited layer in different deposited layer samples.

Sample	Element Content (wt %)		
	Ti	Al	Nb
Single layer sample	74.57	7.58	13.97
Two layers sample	67.26	8.31	22.94
Three layers sample	58.85	9.06	29.55
Four layers sample	54.95	9.26	33.64
Five layers sample	51.91	9.70	37.78
Six layers sample	50.50	9.96	39.12
Seven layers sample	47.73	10.12	42.09
Eight layers sample	45.16	10.44	43.13
Nine layers sample	44.58	10.52	44.75
Ten layers sample	43.48	10.69	45.69
